# Supplementary material for: Are carbon emissions trading and green financial instruments synergistic? -Comprehensive quantitative research based on content analysis
Source: PLoS One. 2024 Mar 7;19(3):e0298601. doi: 10.1371/journal.pone.0298601 (PMC10919601; doi:10.1371/journal.pone.0298601)
Supplement: S1 Data — (ZIP) [file pone.0298601.s001.zip › Data/地级市Z_F工作报告.zip!/┴╔─■/│»╤⌠╩╨/└┤╫╘╣1⁄2╓┌║┼í╛┬φ┐╦╩2╛▌═°í┐_2014.docx]

各位代表：

　　现在，我代表市人民政府向大会报告工作，请予审议，并请各位政协委员和其他列席人员提出意见。

　　一、过去一年工作回顾

2013年，在省委、省政府和市委的正确领导下，在市人大及其常委会和市政协的监督支持下，我们面对极为严峻复杂的经济形势，迎难而上，顽强拼搏，较好地完成了市十届人大一次会议确定的各项任务。

　　--预计地区生产总值增长9.5%，总量突破千亿元大关。

　　--新型工业化实现破题并迈出坚实一步，工业园区和产业集群建设加速推进，一批核心项目成功落地。

　　--“5+1”新城建设突飞猛进，新开工项目177个，完成建筑面积1000万平方米，全市城镇化率提升2.6个百分点。

　　--设施农业提质增效工程成效显著，开始由“量”向“质”的调整升级，设施农业发展站在了一个新的历史起点上。

　　--大小凌河流域综合治理取得突破性进展，水质水量和生态环境明显改善，非法采砂、占河、排污等顽症得到有效控制，“三年大见成效”目标圆满完成。

　　与此同时，其他各项主要经济指标继续保持较快增长，预计完成公共财政预算收入112.5亿元，增长5%；固定资产投资836亿元，增长17%；社会消费品零售总额334.1亿元，增长13.5%；城镇居民人均可支配收入18990元，增长11%；农民人均纯收入9905元，增长14%。

　　一年来，我们以科学发展观统领经济社会发展全局，主要做了以下工作：

　　(一)采取超常措施推进三次产业稳增长按照省委、省政府的总体要求并结合朝阳实际，牢固树立工业强市理念，进一步明确了“工业发展园区化、园区发展产业化、产业发展集群化、集群发展特色化”的工作思路，扎实开展产业集群建设年活动。上海飞和10万台整车组装、北京中智力拓镀膜玻璃、紫陶建材、佰宏钒钛等项目破土动工；金麟超纯铁粉、电容型锂离子电池产业化、北票盛泽页岩油、唯科东明扩建等项目顺利投产；重机、朝重、长峰电源、金达钛业、黑猫炭黑等中心城区老企业“退二进三”搬迁改造扎实推进。产业集群销售收入均增长20%以上。我们还通过落实政府领导包建项目责任制、实施铁选企业电价补贴等措施，帮助企业促开工、渡难关，扭转了工业增长一度出现的下降趋势，规模以上工业增加值增长12.5%，居全省前列。

　　及时出台拉动建筑业和房地产业发展的政策措施，主动为71个有遗留问题楼盘完善手续、办理房证，举办市县两级房交会10余次，完成建筑业施工面积2100万平方米，实现产值380亿元；完成房地产开发1920万平方米，实现商品房销售额220亿元。

　　积极应对低温寡照、干旱少雨等自然灾害，粮食产量连续三年超25亿公斤。通过秸秆生物反应堆技术推广、农产品质量监管、弃管闲置棚恢复利用等措施，设施农业实现产值155亿元，新增节水滴灌面积40.5万亩、畜禽标准化养殖小区680个。

　　启动实施服务业建设年活动，通过夜经济、农超对接、会展经济等措施，服务业保持快速发展。新增贷款、直接融资跃上“双百亿”台阶，盛京、锦州银行在我市设立分行，朝阳银行抚顺分行挂牌营业。首届文化旅游节、第二届化石节成功举办，文化旅游体制机制不断创新，我市荣获中国“化石之都”和“观赏石之城”称号。

　　(二)强力推进项目建设和改革开放实施亿元以上重点项目248个、10亿元以上重大项目36个，完成投资520亿元。全市瞩目的京沈高铁朝阳段征地拆迁工作全面展开，国电热电联产项目获正式核准。经过近100天的奋战，总投资7.2亿元的燕山湖集中供热项目建成投入使用，供热面积达506万平方米，由两家电厂集中供热的市区供暖格局逐步形成。

　　坚持“精干、节俭、高效、廉洁”的原则，组织开展了赴香港、台湾、上海、唐山、呼和浩特、晋江等14次招商，洽谈推进项目300多个，项目落地率达60%以上。成功引入9个工业地产项目，开工建设厂房210万平方米。域外引资增长18.3%；实际利用外资2.16亿美元，增长20%；出口创汇5亿美元，增长25.6%，其中农产品出口突破5000万美元，实现了翻两番。

　　城区财政管理体制改革顺利实施，税收征管秩序不断规范，市区两级发展积极性得到有效调动。主动向城区下放经济管理权限，进一步理顺了朝阳工业新区、燕都新区管理体制机制。深化行政审批制度改革，优化审批流程，政府投资、工业、房地产类项目审批时限全部压缩到60个工作日之内。按照“一委一办一中心”要求，公共资源交易中心组建运行。

　　(三)加大城乡建设力度“5+1”新城起步区初具规模。燕都新区“四横四纵”路网框架初步形成，南京中贸商业城项目成功落地，燕都大桥竣工通车，新区安居工程、城市规划展览馆、中水供热制冷项目等相继建成；凤凰组团和五县(市)新城一批重点项目建设也取得新进展。城中村、城边村改造大面积实施，小城镇建设进一步加快，多点推进城镇化格局已经形成。

　　老城区改造深入实施。中心城区控制性详细规划编制加快推进。新建和改造市县两级城市道路130万平方米、排水管网86公里。大凌河两岸、仿古一条街及城区主要街路亮化工程全面实施，学府广场和高速南北口绿地、燕阳山南路和北大桥头拓宽改造、哨口大桥亮化等工程顺利完成，气化朝阳天然气主管道铺设全线贯通。“朝阳-大连-上海”航线正式开通。市容环境集中整治深入展开，环卫管理模式和运行机制得到理顺，城市管理更加有序。

　　不断提高农村公路养护管理水平，维修改造普通公路810.7公里，总投资创历史新高。308个行政村环境治理任务全面完成。

　　扎实推进青山、碧水、蓝天三大生态工程建设。“两退一围”任务超额完成，植树造林140.4万亩，水土保持生态治理91.2万亩，草原沙化治理58万亩，我市荣获全国绿化模范城市称号。大小凌河治理攻坚战深入实施，城市段防洪景观工程成效显著，干流河滩植被覆盖率已达81%；32个水污染治理项目和市区污水调水工程竣工并投入运行，中心城区污水实现零排放。水源热泵和中水回用技术推广、公交车油改气(电)等工作扎实推进。

　　(四)大力发展县域经济和城区经济经过一年的努力，县域经济主力军作用进一步凸显，城区经济焕发出新活力，七个县(市)区呈现你追我赶、竞相发展态势。

　　北票粉末冶金、页岩油两大产业集群建设初具规模。台吉大街全线通车，华鹏盛典、宏达地产等新城项目启动建设。大黑山晋升国家自然保护区和省沟域旅游示范区，温泉小镇项目基本建成。北票市荣获全国日光温室越夏番茄第一县称号。

　　凌源汽车、玻璃两个产业集群核心项目成功引入，为园区爆发式增长奠定重要基础。滨河新城起步区框架全面拉开，城区段内河实现蓄水，永利广场、兴隆大家庭等商业综合体加快推进。凌源市荣获省级森林城市称号，青龙河核心区晋升国家自然保护区。

　　朝阳县新县城建设取得重大进展，行政中心即将搬迁。以“6+4”产业为支撑的有色金属产业集群初见规模。温氏集团生猪养殖项目顺利投产，小凌河26.7公里滨河路实现贯通。朝阳县荣获中国鲜食枣第一县称号，并被确定为全省一县一业示范县。

　　建平县有望连续三年成为全国粮食生产先进县。膨润土深加工产业集群规划建设加速起步，红山新城建设扎实推进，行政中心和两所高水平中学项目全面建成。红山玉三期项目启动实施。建平县荣获全国土地节约集约利用模范县称号。

　　喀左凌河治理、农村环境整治、校车管理等工作走在全市前列。冶金铸锻产业集群不断壮大，紫陶建材产业被列入全国“百城万亿”增长计划重点培育项目。河南新城规划展览馆等项目全面竣工。喀左县荣获国家农产品出口安全示范县和省级森林城市称号。

　　双塔金属新材料产业集群建设强势起步，一批核心项目建设取得明显进展。城区拆迁强力推进，开创了按法规、依程序、高效率拆迁的新纪录。孟克、榆树林、中山三大区片城中村改造快速推进。双塔区被评为全省发展服务业先进城区。

　　龙城新能源电器、装备制造、食品加工三大产业集群建设齐头并进，一批新项目为工业新区的崛起增添了新动力。城中村、城边村改造任务超额完成。新天地商贸文化城全面竣工，豪德广场营业额突破30亿元。

　　(五)加强以民生为重点的社会建设统筹城乡就业，完成实名制就业6.9万人。社保覆盖面进一步扩大，企业基本养老保险扩面征缴百日行动全面展开，城乡低保规范化管理得到加强。

　　积极打造“社区十分钟便民服务圈”，开展了社区老年人日间照料等9项便民服务，20个首批示范社区建设取得明显成效。

　　投入资金9.5亿元，全面完成了市政府承诺的10件实事，其中，实施老旧小区改造200万平方米，新建安全饮水工程337处，企业退休人员工资、城乡低保、城镇居民基本医疗保险待遇水平进一步提高，农村低收入贫困人口年人均纯收入达到2500元以上。新建改扩建全市城区中小学18所，大班额问题得到有效缓解。

　　各项社会事业健康发展，社会管理不断加强。新建改扩建标准化幼儿园24所，燕都新区一高、一中分校开工建设。特色高中教育喜获丰收，中职教育活力增强，高等教育稳步攀升。县级公立医院综合改革、乡镇卫生院实行基本药物制度、乡村卫生服务实施一体化管理全面铺开，市卫校、四院、康宁医院、中心医院外科病房楼、二院综合楼建设全部完成，新增床位2400张。企业人才培养引进、国内外企业并购等工作取得新进展，高新技术产品增加值增长20%。牛河梁国家考古遗址公园获国家批准通过，第二届群众文化节成功举办，电视剧《老家门口唱大戏》在央视播出。十二届全运会男子排球比赛和火炬传递任务圆满完成，得到省政府和国家体育总局排管中心的高度评价。国土资源管理水平和保障能力显著提高。稳妥推进诉访分离，信访秩序持续好转。加强社会治安综合治理，维护稳定、安全保卫、道路交通秩序整治取得明显成效。中心城区烟花爆竹限时燃放管理全面实行。持续开展隐患排查和整改，安全生产形势总体平稳。民族团结进步事业取得新成绩。双拥共建成果进一步巩固，我市顺利通过省双拥模范城中期检查验收。

　　人口和计划生育、质量监督、食品药品安全、工商、物价、邮政、档案、住房公积金、应急管理、人防、外事侨务和涉台工作，以及老年人、妇女儿童、残疾人权益保障等工作也取得了新成绩。

　　(六)加强民主法制和政府自身建设坚持依法行政，自觉接受人大及其常委会的法律监督和工作监督，主动接受政协的民主监督，积极支持各民主党派、工商联、无党派人士和人民团体参政议政，共办理人大代表建议68件、政协提案192件，满意率均达到98%以上。

　　加强政府精细化管理，建立市长例会等制度，出台了一系列改进办公流程、转变运行机制、提高工作效率的新举措，政府管理水平进一步提高。市政府班子累计下基层解决实际问题4000多件，组织各项拉练、督查160多次。深入搞好行政监察和审计监督，政府投资项目资金审减额达2.7亿元。通过开展软环境整治百日行动、压缩“三公”经费支出、推进涉企收费大检查，政府工作越来越得到人民群众的理解和支持。

　　各位代表！回顾过去一年，我们与全市人民携手奋战，艰苦跋涉，沉着应对各种风险挑战，在加快发展的征程上留下了坚实足迹。所有这一切，饱含着省委、省政府的关怀和厚爱，深系着市委的坚强领导和鼎力支持，倾注着市人大、市政协的心血和汗水，也凝结着全市各族人民的聪明才智和辛勤劳动。在此，我代表市政府，向在各个领域和岗位上辛勤耕耘的全市各族人民，向关心支持朝阳发展的老领导、老同志，向给予我们监督与支持的人大代表、政协委员、各民主党派、工商联、无党派及社会各界人士，向情系朝阳、支持朝阳的驻朝中省直单位、解放军指战员和武警官兵，表示崇高的敬意和衷心的感谢！

　　在总结工作的同时，我们也清醒认识到，制约全市发展的结构性矛盾和问题尚未根本解决。突出表现在：工业化和城镇化水平低，产业结构单一，服务业发展缓慢，对外开放度不高，科技创新能力不强，城区经济薄弱，生态环境还很脆弱，民生尚处于低水平阶段。特别是由于个别干部责任意识不强、管理不到位、工作失职，导致安全生产等一些工作抓得不实、成效不明显，“四风”问题和消极腐败现象依然存在。对此，我们一定高度重视，并采取有力措施认真加以解决。

　　二、全面深化改革

各位代表，党的十八届三中全会做出了全面深化改革的重要决议。今年及今后一个时期，我们要按照省委、省政府的改革部署，并结合朝阳实际，积极稳妥地推进以下改革：

　　(一)推进土地集约化经营。加快发展以农民合作社为主的新型农业经营主体，建立多元统一的农村土地流转市场，全力开展以规模经营、农村金融创新为主的农村综合改革。

　　(二)推进户籍制度改革。进一步落实“一元化”户籍登记管理制度，搞好城区流动人口服务管理创新，跟进配套措施，有序推进农业转移人口市民化，让进城落户的农民逐渐享受市民待遇。

　　(三)推进国有企业改革。稳妥推进重点国有企业走混合所有制企业发展路子。认真执行国有资本经营预算制度。对部门开办的小微企业要彻底清理，尽快退出国有。

　　(四)推进公用事业改革。积极推进包括自来水、燃气、供暖、市政、园林、环卫、公交、旅游在内的各项改革，规范公司治理，深化三项制度改革，加强服务质量、价格等监管，建立起适应市场经济的体制机制。

　　(五)推进市场准入制度改革。把注册资本实缴登记制改为认缴登记制，取消企业和个体工商户年检验照，并通过更为有效的扶持政策，促进民营经济和中小微企业等各类市场主体成长。

　　(六)推进各类市场体系建设。搞好城区“8+2”商贸市场和农村流通体系建设。加大金融、人才、技术等市场建设。

　　(七)推进中介机构改革。建立政府主导和市场相结合的中介服务价格形成机制，营造公平竞争的市场环境，防止个别中介机构垄断市场。政府部门一律不得办中介机构，已开办的要脱钩。加强中介机构信用管理，推行不良行为记录公示制度。

　　(八)推进价格改革。完善反映市场供求状况、资源稀缺程度和损害环境成本的价格形成机制。合理调整天然气价格，推行阶梯价格。

　　(九)推进财税体制改革。建立现代财政制度，完善预算管理制度，建立政府债务风险管控机制。完善城区财政管理体制，强化财源建设和税源监控。健全政府非税收入征收管理体制。调整和优化财政支出结构，压缩一般性支出。完善国库集中收付制度改革，加强预算绩效管理，推进预算公开。

　　(十)推进金融服务创新。积极引进域外金融机构，加大企业挂牌上市推进力度。支持设立产投、创(风)投基金。深入开展“助保贷”、“采购贷”等金融服务。推进建平农商行组建试点工作。

　　(十一)推进科技体制改革。重点发挥现有高新技术企业创新骨干作用，鼓励企业建立研发中心、工程中心和省级以上重点实验室，支持中小企业自主创新。建立科研院所与企业协同创新机制，促进科研成果转化。

　　(十二)推进教育综合改革。均衡配置各类教育资源，全面实行中小学阳光平行分班制度，禁止跨学区招生，不设重点校和重点班，继续实行区域内校长和骨干教师交流轮岗制度。扩大省级示范高中定向招生比重。整合中职学校专业资源，推进产教融合、校企合作。探索符合国家和省要求、符合未来发展趋势、符合朝阳实际的高等教育和成人教育新途径。

　　(十三)推进医药卫生体制改革。深化基层医疗卫生机构综合改革，加快公立医院改革。全面实施城乡居民大病保险，推进城乡居民基本医疗保险制度整合。建立全市药品、耗材、医疗器械统一招标采购平台和区域卫生信息平台。启动实施一方是独生子女的夫妇可生育两个孩子政策。

　　(十四)推进文化事业和文化产业不断发展。扶持公益性文化事业，发展具有朝阳地域特色的文化产业。推进新华书店体制改革。繁荣文艺创作，培育演艺市场。

　　(十五)推进生态文明制度建设。在碧水工程上，严格实施“河长制”考核，完善基层管护体系建设，对污水处理设施实行在线监控，消除污水直排现象，对达不到要求的，扣缴生态补偿金并限期整改。在青山工程上，加大环境恢复治理保证金收缴力度，严格控制新设立采矿权。在蓝天工程上，实施各县(市)区空气质量考核制度，对达不到要求的，扣缴生态补偿金。

　　(十六)推进对外开放。建立招商项目落地协调责任机制，提高项目落地率。构建出口企业服务平台，促进企业直接出口，打造全省农产品出口基地。建立激励机制，引导企业走向国际市场。

　　(十七)推进新区体制创新。围绕提高工作效率、加快项目建设和产业集聚，抓好朝阳工业新区和燕都新区管理体制机制创新。

　　(十八)推进政府职能转变。重点改变机关内部机构设置和职能配置。面向基层、面向实际、面向生产力设置机构、配置人员，改变机关为机关服务的现象，改变干部无用武之地的现象。完成卫生计生机构整合、工商质监划归地方、食药监管理体制完善等工作。

　　(十九)深化行政审批制度改革。进一步下放审批权限，缩短审批时限，凡规定市级投资主管部门核准的审批和备案管理权限，原则上全部下放到各县(市)区。

　　(二十)改进绩效考核制度。合理确定考核内容和指标，除继续考核主要经济指标外，要加大资源能源消耗、环境损害、生态效益、产能过剩、科技创新、政府性债务、安全生产等指标权重。

　　(二十一)推进事业单位改革。积极推进行政类、生产经营类事业单位改革，搞好公益类事业单位法人治理结构试点，对职能萎缩、弱化和职能相近的公益类事业单位进行机构编制调整。

　　(二十二)强化权力运行制约和监督。发挥市场在资源配置中的决定性作用，公共资源交易项目必须全部进入中心，做到应进必进。建立重大决策事项社会稳定风险评估制度，构建决策科学、执行坚决、监督有力的权力运行体系。加强行政监察和审计监督，完善惩治和预防腐败体系。围绕反对“四风”，加快体制机制改革和建设，完善政府作风建设常态化制度。

　　三、2014年工作安排

今年政府工作的总体要求是：全面贯彻落实党的十八大、十八届三中全会精神，按照市委九届六次全会暨经济工作会议的部署，坚持稳中求进、改革创新总基调，深入实施“四化三大”战略，全面推进经济、政治、文化、社会、生态文明建设，实现经济持续健康发展和社会和谐稳定。

　　主要预期目标是：地区生产总值、公共财政预算收入均增长10%， 固定资产投资增长20%，社会消费品零售总额增长14%，城镇居民人均可支配收入和农民人均纯收入分别增长12%和13%。

　　(一)加快推进新型工业化进程开展园区基础设施和产业集群建设年活动，围绕一批有产业基础的老企业搬迁改造，抓提档升级；围绕提升资源型企业加工度，抓产业延伸；围绕战略性新兴产业和高新技术产业，抓增量拓展，尽快形成工业多点支撑格局，实现“一年强基础，三年见成效，五年建成产业链完整、园区功能完备、支撑地区经济发展的支柱产业”目标。

　　完善各类园区规划、基础设施和配套功能，推进起步核心区“七通一平”，搞好公共展示、技术研发检测、公共信息、物流、生活等多重服务平台建设。

　　加快推进产业集群核心项目建设。新能源电器产业集群要争取建成8-10条电容型锂离子电池生产线，积极引进台湾瑞能电动车等项目；装备制造产业集群要加快推进重机、朝重、黑猫炭黑等老企业搬迁改造；食品加工产业集群要瞄准国内外品牌农事企业抓敲门招商；双塔金属新材料产业集群要积极推进金麟超纯铁粉、天翼国基超纯合金、金达钼钛、朝柴搬迁改造等项目；北票粉末冶金、页岩油产业集群要加快实施富贵鸟30万吨铸锻件、盛泽和北煤海亮页岩油等项目；凌源汽车、玻璃产业集群要全力推进整车组装、镀膜玻璃等项目；朝阳县有色金属产业集群要抓好新都黄金搬迁改造、兄弟资源不锈钢等项目；建平膨润土、金属新材料产业集群要继续推进永同昌膨润土和旗盛、纳鑫粉末冶金等项目；喀左冶金铸锻、紫砂产业集群要抓好金工科技钒钛、紫陶建材等项目。要发挥好这批核心项目的“孵化器”作用，积极引进上下游企业，延伸产业链条，提升产业丰厚度。北票粉末冶金、朝阳县有色金属产业集群销售收入达到300亿元，其他产业集群销售收入增长20%以上。

　　深入推进工业调结构、促转型。加快运用信息技术改造和提升传统工业。继续支持凌钢、鞍凌钢、燕山湖电厂等企业搞好生产经营。新增销售收入超10亿元企业2户、超亿元企业15户，规模以上工业增加值增长12%。完成省下达的并购国内外企业、节能降耗和淘汰落后产能任务。高新技术产品增加值增长20%以上。

　　(二)进一步提升城镇化水平开发建设“5+1”新城、推进城中村改造、抓好特色小城镇建设、加快老城区改造，是朝阳向新型城镇化迈进的重要举措。我们要继续坚持高标准建设、高水平开发、高速度改造，积极推进以人为核心的城镇化，确保完成建筑施工面积2100万平方米，城镇化率提高2个百分点以上。

　　启动实施燕都新区辽西文创城、雅氏养老中心等项目，积极推进中贸商业城、万宇佳业等项目，全面完成商务大厦、规划展览馆布展等项目，形成起步区城市形象；其他新城要加快推进重点项目建设，增强产业发展、公共服务、吸纳就业、人口集聚功能。“5+1”新城新开工面积1000万平方米。继续搞好凤凰组团开发建设。

　　坚持中心城区改造先行、市区高速公路出入口改造先行、临主要街路改造先行，加快实施哨口、八里堡、八宝、西大营子、七道泉子等城中村改造，完成改造面积400万平方米以上。

　　按照有产业支撑、省际相邻、凌河沿岸、临交通干线等不同类型，每个县(市)重点推进3-5个特色小城镇建设，新开工面积200万平方米以上。

　　加大老城区改造力度，新建和改造市县两级城市道路40万平方米、排水管网45公里。继续开展市容环境集中整治行动。

　　(三)大力发展现代农业牢牢抓住、紧紧抓好“三农”工作，不断加大强农惠农富农政策力度，让农业更强、农村更美、农民更平等地分享现代化成果。

　　深入推进设施农业提质增效工程，围绕建成国家现代设施农业示范区，稳步扩大面积，大力推广秸秆生物反应堆技术和草腐菌试验示范，搞好结构调整、技术服务、市场物流、农产品质量监管等工作，培育无公害绿色有机品牌。新增节水滴灌面积95万亩。抓住全省实施千万亩经济林建设工程机遇，新增经济林面积50万亩。

　　在每个县(市)规划建设一个农产品和食品加工园，积极推进新加坡周氏集团、蒙牛集团等一批重点项目落地，年内力争再引进2-3个投资10亿元以上的重大农产品加工项目。

　　加强农村公路养护管理，推进村屯环境综合整治，搞好美丽乡村建设。坚持开展“大禹杯”竞赛活动，完成水土保持生态治理面积50万亩。完善突发事件预警信息发布系统建设，科学开发云水资源。

　　(四)加快发展城区经济和服务业进一步加大对城区经济发展的支持力度，两区主要经济指标增幅高于全市平均水平2个百分点以上。

　　继续开展服务业建设年活动。加快推进各县(市)“1+2”服务业集聚区建设，打造双塔生活资料物流园区、龙城生产资料物流园区，启动建设燕都新区、凤凰休闲旅游服务业集聚区。大力发展电子商务、信息、金融、服务外包、养老、会展等现代服务业，打造朝阳夜经济“升级版”。

　　加快创新文化旅游体制机制，整合活化四大文化旅游资源，编制旅游业发展专项规划，搞好各类文化产业园区建设，积极推进温泉旅游、沟域旅游和乡村旅游等系列精品项目建设，启动培育旅游地接市场，把文化旅游业逐步打造成一个支柱产业。

　　继续保持房地产业健康发展，房地产开发面积达到1600万平方米，房地产投资额和商品房销售额均增长15%以上。

　　(五)搞好招商引资和项目建设树立大开放、大招商理念，坚定不移地推进“精干、节俭、高效、廉洁”招商，每月至少组织一次专项招商活动，域外引资、实际利用外资和出口创汇均增长20%。抓好工业地产招商，每个县(市)区新建工业地产厂房达到30万平方米以上。

　　深入落实市政府领导包建项目责任制，实施投资亿元以上重点项目300个，完成投资600亿元以上。新增贷款110亿元。坚持节约集约利用土地，抓好未利用地开发、城乡建设用地增减挂钩和低效土地利用，实现建设用地征收与转用审批1500公顷。

　　(六)加强城乡基础设施建设全面完成京沈高铁朝阳段征地拆迁任务，为项目开工建设提供保障；启动朝盘、牛河梁至赤峰两条高铁连接线前期工作，打造朝阳高铁区域枢纽地位。开工建设国电热电联产项目，完成厂区“五通一平”和主厂房建设。配合省里搞好引白入朝供水工程，加快推进锦白铁路扩能改造、义县-叶柏寿-凌源电气化复线改造、500千伏利州输变电等项目。完成燕山湖-白石水库滨河路建设，开工建设燕阳山南路大桥。推进天然气管线铺设向县镇、企业延伸，加快实现气化朝阳目标。

　　(七)深入实施青山、碧水、蓝天三大生态工程以燕阳山公园、凤凰山迎城面生态修复、城区高速口周边迎面山绿化为重点，启动实施14.4万亩的中心城区环城绿化带建设，争取三年内全部建成，年内完成5万亩绿化任务。新增植树造林100万亩，全民义务植树900万株。

　　按照巩固、扩面和提标的要求，编制凌河流域综合治理开发总体规划，继续打好流域深度整治攻坚战，加快形成生态带、旅游带和城镇带，如期完成大小凌河治理目标。拓展中水回用市场，实现污水治理工程稳定运行。利用社会力量和资金，实施大凌河燕都新区段音乐喷泉、水幕电影和凌河湾湿地等项目建设。

　　继续抓好工业企业脱硫脱硝除尘、公交车油改气(电)、重金属污染防治、扬尘治理等工作。建设4座空气自动监测站，实行日监测发布，空气质量排名进入全省前列。

　　(八)全力抓好民生工程民生问题永远是我们的工作重点。我们要将其贯穿于政府的一切工作之中，让人民群众得到更多实惠，感受更多的温暖和幸福！

　　一要抓好扩大就业工程。推进创业型城市创建工作，新增实名制就业4.8万人，城镇登记失业率控制在4%以内。构建和谐劳动关系，严厉打击侵害劳动者特别是农民工权益的行为。

　　二要抓好社会保障工程。扩大各类社会保险覆盖面，新增“五险”扩面3.5万人次。继续对城乡低保和企业退休人员基本养老金进行提标，进一步堵塞城乡低保管理漏洞。全面实行城镇基本医疗保险门诊大病、慢性病即时结算。

　　三要抓好扶贫救助工程。完成37个综合扶贫示范村建设和500户移民搬迁任务，确保10万农村低收入贫困人口稳定脱贫。推行城乡医疗救助一站式即时结算服务管理模式。对独生子女失独伤残家庭实行救助。解决10.2万农村人口饮水安全问题。

　　四要抓好城乡安居工程。搞好热源整合，中心城区南部集中供热面积达到1200万平方米。实施市县两级老旧小区改造240万平方米，继续抓好一批“暖房子”工程。启动村庄亮化工程建设，为1028个行政村安置路灯。

　　五要抓好教育助学工程。新建改扩建标准化幼儿园16所，完成200所中小学达标任务，力争3所高中进入省特色普通高中实验学校。继续解决中心城区和县城中小学大班额问题。加强校车配备和管理，保障中小学生交通安全。做好留守儿童管理工作。

　　六要抓好医疗惠民工程。搞好全市急救网络指挥平台建设，实现院前急救无盲区。将新农合人均筹资标准提高到400元。大力开展减盐限盐活动。

　　七要抓好“社区十分钟便民服务圈”建设工程。以居住地为圆心，以步行不超过10分钟为半径，让群众就近就便地享受到生活、医疗、就业、购物、出行等服务，实现全市城市社区全覆盖。

　　八要抓好文体惠民工程。继续搞好凌河沿岸群众文化活动带建设，举办牛河梁红山文化节和群众文化节等活动，开展送戏下乡400场，建成社区书屋62个，完成燕都图书馆建设。积极开展国民体质监测，新建一批农民体育健身工程和公共篮球场，改造修缮社区健身路径。

　　九要抓好养老助残工程。实行高龄补贴扩面提标。实现中心城区社区老年人日间照料室建设全覆盖，县(市)覆盖面达到50%。为全市符合条件的听力残疾人免费适配助听器。加快推进县级殡仪馆基础设施设备改造更新。

　　十要抓好消费安全工程。加强食品药品监管，提升农产品检验监测水平，严厉打击制售假冒伪劣商品行为。

　　(九)创新和提高社会治理水平稳妥推进信访改革，完善矛盾纠纷化解机制，积极推进人民调解、行政调解、司法调解工作。落实企业主体责任，搞好尾矿库集中整治，坚决遏制重特大事故发生。推进平安朝阳建设，创新立体化社会治安防控体系，防范和打击各类违法犯罪，积极争创全国综治工作优秀市。强化国防教育和民兵预备役、人民防空等工作，深入开展双拥共建活动，不断开创军民融合式发展新途径。贯彻落实民族宗教政策，支持少数民族和民族地区加快发展。做好新一届社区居委会换届选举工作。搞好全国第三次经济普查。

　　(十)加强政府自身建设我们要自觉接受人大的法律监督、工作监督和政协的民主监督，坚决执行人大及其常委会的决议、决定，认真办理人大代表议案、建议和政协提案，坚持依法行政，强化行政执法监督，深化政务公开，建设法治政府。

　　要创新行政管理方式，推进精细化管理，提高工作决策指导能力和解决实际问题能力，建设创新政府。

　　要倡导事不过夜、事无巨细、事必躬亲和干事、干练、干净的工作作风，深入实际，走进矛盾，敢于负责，勇于担当，建设高效政府。

　　要廉洁从政，坚持厉行节约，反对铺张浪费，建设廉洁政府。

　　我们一定要通过政府自身建设的强化，通过服务型政府的塑造，让全市人民看到一个讲诚信、懂规矩、守纪律的政府团队，看到一支勤奋、廉洁、公道、正派的公务员队伍。

　　各位代表！带着一年的汗水和收获，我们迈进了充满激情挑战的2014年。新的征程不会是一片坦途，但朝阳不能停步，朝阳人不能停步，朝阳求发展、求突破、求振兴的梦想不能停步。我们将在市委的坚强领导下，团结和带领全市各族人民，继续以夙兴夜寐、奋发有为的精神状态，全面完成本次会议确定的各项任务，向着未来，向着胜利，向着全面建成小康社会的目标奋勇前进！
